# Case report: Robust response of metastatic clear cell sarcoma treated with cabozantinib and immunotherapy

**DOI:** 10.3389/fped.2022.940927

**Published:** 2022-10-06

**Authors:** Rakefet Sidlik Muskatel, Nir Pillar, Jeremy Godefroy, Michal Lotem, Gal Goldstein

**Affiliations:** ^1^The Dyna and Fala Weinstock Department of Pediatric Hematology Oncology, Hadassah Hebrew University Medical Center, Jerusalem, Israel; ^2^Department of Pathology, Hadassah Hebrew University Medical Center, Jerusalem, Israel; ^3^Hadassah Hebrew University Medical Center, Nuclear Medicine Institute, Jerusalem, Israel; ^4^Center for Melanoma and Cancer Immunotherapy, Hadassah Hebrew University Medical Center, Sharett Institute of Oncology, Jerusalem, Israel

**Keywords:** clear cell sarcoma, immunotherapy, cabozantinib, MET inhibitor, EWSR1-ATF1 translocation

## Abstract

Clear Cell Sarcoma (CCS), also referred to as malignant melanoma of soft parts, is a rare and aggressive malignant tumor. It comprises 1% of all soft tissue sarcomas and is known to be radio- and chemotherapy resistant. CCS shares morphological and immunohistochemical features with malignant melanoma, including melanin biosynthesis and melanocytic markers. However, it is distinct for the presence of EWSR1-ATF1 translocation which activates MITF transcription factor. We report here of an aggressive case of CCS in a 9-year-old patient, which demonstrates the critical role of molecular analysis in the diagnosis and treatment of uncommon cancer variants in the era of personalized medicine. The EWSR1-ATF1 translocation induces pathological c-Met activation, and so, following unsuccessful CTLA4 and PD-1 blockade immunotherapy, the child received cabozantinib, a small molecule tyrosine kinase inhibitor, with the intent to block c-Met oncogenic effect. In parallel, active immunization, using hapten di-nitrophenyl modified autologous tumor cells was administered with monotherapy PD-1 inhibitor nivolumab. Under this “triplet” therapy, the patient attained an initial partial response and was progression-free for 2 years, in good performance status and resumed schooling. Based on our observation, cabozantinib can be used as an effective and potentially life-prolonging treatment in CCS. We suggest that priming the child’s immune system using her autologous tumor and combating T cell exhaustion with PD-1 blockade may have synergized with the targeted therapy. Combining targeted and immunotherapy is a rapidly growing practice in solid tumors and provides a glimpse of hope in situations that previously lacked any treatment option.

## Introduction

Clear Cell Sarcoma (CCS), is a rare and aggressive malignant tumor. It was first defined by Enzinger (1) in a series of 21 cases, collected over 25 years. He then identified a distinct type of sarcoma that arises mostly from tendons and aponeuroses of extremities. Due to the presence of melanin pigment, immunohistochemical staining for S-100 and other melanocytic markers, CCS resembles melanoma and was granted the name of “melanoma of soft parts” (2). The disease, comprising only 1% of all soft tissue sarcomas, appears mostly in extremities (3), while head and neck CCS are rarer, and comprise only 1–2% of reported cases (4). Complete surgical excision and a small tumor size are the main prognostic factors, since there is poor response to radio- and chemotherapy (5–8). The prognosis of head and neck cases, especially in a site that does not allow complete resection, is grim (5, 9). Reviewing the literature, only few studies and reports of head and neck CCS cases are found (3, 10–13). Metastases are common at presentation, or appear as late relapses- 30 and 63%, respectively (14, 15). Lymphatic spread is common, as well as lung metastases (16). The 5-years survival rate of metastatic CCS is only 20% [5–7]. CCS is characterized in most cases by the t(12;22)(q13;q12) translocation, that involves the Ewing sarcoma gene (*EWS*) on chromosome 22, with the cAMP regulated transcription factor *ATF1* on chromosome 12, a member of the CREB family (17–20). This translocation has a direct impact on the MITF pathway resulting in c-Met aberrant expression (21). This abnormal expression plays, most probably, an important role in the malignant transformation of CCS progenitor cell. Hence, c-Met can be the molecular target of novel directed therapy for CCS. Herein, we present an effective treatment for metastatic clear cell sarcoma by targeting c-Met with cabozantinib, a semi-selective signal blocker.

## Case presentation

**FIGURE 1:**
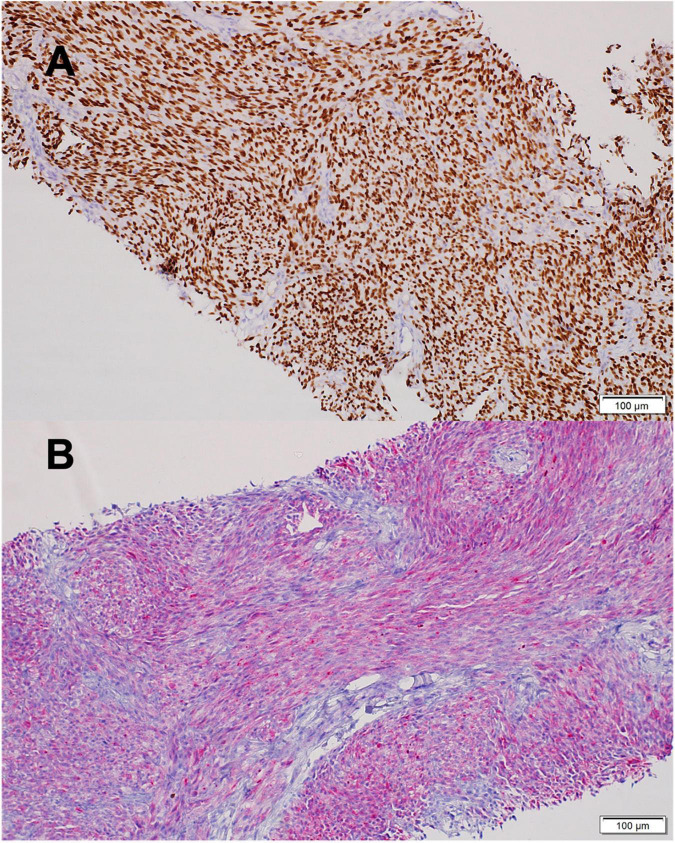
Clear Cell Sarcoma histology. **(A)** SOX10 positive staining. **(B)** MART1 positive staining. Scale bars 100 mm. Images were acquired by brightfield Olympus BX53 microscope with X20 air objective. (Olympus Life Science, Waltham, MA, United States), and Llumins 5MP Bright Field Camera (Llumins, Johnannesburg, South Africa). Software- Llumins ToupView (Llumins).

A 9-year-old girl was evaluated for a bleeding mass in her left external auditory canal. Initial pathology report revealed a malignant tumor composed of hyperchromatic spindle and ovoid cells, focally demonstrating pigment, with areas of necrosis. Vascular invasion was demonstrated. The tumor cells stained for pan-melanoma markers including MART-1, HMB-45, and SOX-10 (Figure 1). The findings were compatible with malignant melanoma, pigmented Schwannoma or CCS. Imaging studies revealed a 3 × 2 cm mass extending and invading the mastoid bone. The child underwent left mastoidectomy and lymph node sampling. The tumor mass was only partially resected and on CT scan 4 weeks later a recurring mass was revealed with new enlarged lymph nodes at the posterior cervical and retro-auricular chains. The rapid, aggressive course of the disease raised the concern that another surgical procedure will only delay systemic therapy. A combination of CTLA-4 and PD-1 blocking antibodies (ipilimumab and nivolumab) was selected based on case reports of benefit in CCS and immune responsiveness of MITF*^high^* melanomas (22, 23). After four courses of therapy, imaging showed new 7-mm lung metastases and enlargement of the cervical lymph nodes. Fresh tissue was retrieved to establish a primary autologous tumor cell culture and chemotherapy with weekly carboplatin was administered to prevent fast, uncontrolled growth of the disease that proved to be immune checkpoint non-responsive. Thereafter, the patient received eight doses (every 3 months) of intra-dermal injections of 25 × 10^6^ irradiated autologous tumor cells admixed with the hapten di-nitrophenyl to prime an initial tumor-specific immune response. This treatment was given based on histologic resemblance to malignant melanoma, and previous encouraging results (24), especially with combination of check-point inhibitor (25).

Genome analysis (FoundationOne^®^CDx, Foundation Medicine, Cambridge, MA) revealed EWSR1-ATF1 translocation, which finalized the diagnosis of CCS.

**FIGURE 2:**
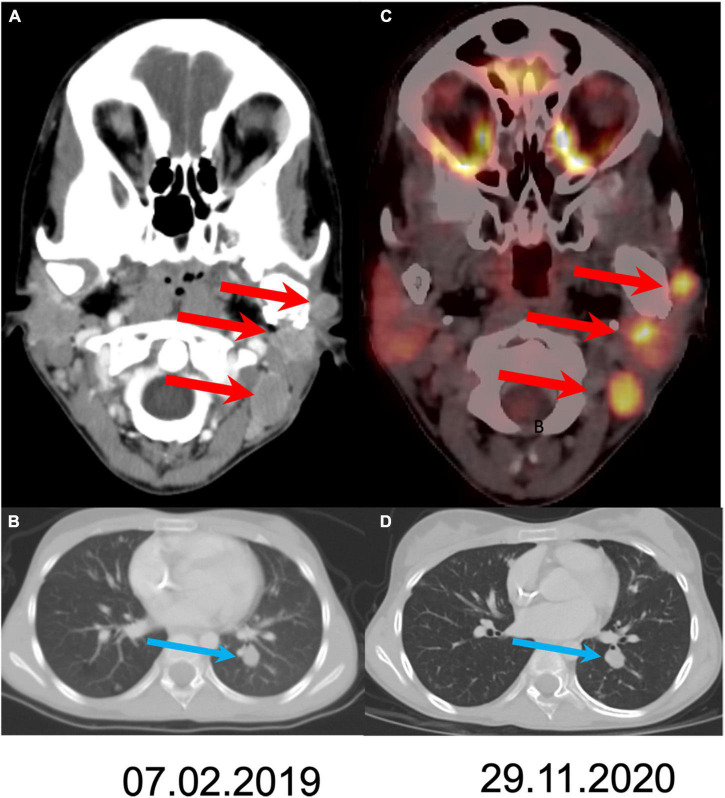
Comparison of disease extent at the initiation of treatment and after 21 months of follow-up. Axial CT slides at staging **(A,B)** show three metastases (arrows) in the left neck and lung spread. Follow up after 21 months shows stable disease both in the neck (**C**; fused PET/CT axial slide) and in the lung **(D)**.

At this point cabozantinib 40 mg/day was started. Dose reductions were made to reach a level that allowed the child to resume daily activity. The treatment was given continuously for 24 months, with a daily dose of 40–20 mg/day. The patient presented with several adverse effects (classified by the CTCAE criteria): intermittent abdominal pain (grade 1) accompanied by moderate anorexia (grade 2) and weight loss, mild hair whitening, and hypothyroidism (grade 2), which are known adverse effects of cabozantinib treatment. Imaging studies showed a mild decrease in size of her lung and cervical lymph nodes, and no disease progression for 24 months-consisted with stable disease by RESICT criteria (Figure 2). The patient has a Lansky performance score of 100, and resumed schooling. Unfortunately, after 2 years, disease progressed again. Once the disease escaped therapy, it progressed rapidly, until the patient’s death.

## Discussion

This case is the first known report of successful stabilization of an aggressive metastatic clear cell sarcoma, under the administration of targeted multi kinase inhibitor and immuno-therapy for 24 months.

This case emphasizes the importance of genomic molecular analysis in the diagnosis and treatment of rare tumors. The EWSR1-ATF1 translocation established a clear distinction between CCS and malignant cutaneous melanoma, and navigated our team toward finding a novel treatment for this patient. EWSR1-ATF1 translocation replaces the kinase-dependent regulatory region of ATF1 with the N-terminal of EWSR1. This chimera results in an oncoprotein that mimics Melanocytic Stimulating Hormone/CREB signaling pathway and aberrantly activates MITF transcription factor (21). The MITF-regulated genes play a role in oncogenesis by activating the c-Met gene (26), which encodes a receptor tyrosine kinase that is normally expressed on stromal and mesenchymal cells and mediates signaling from hepatocyte growth factor. Few studies showed that c-Met is aberrantly expressed in several malignant tumors, such as small cell lung cancer and sarcomas (27, 28), and affects cell survival, growth, invasion and metastasis (29, 30). Previous studies showed that in CCS, EWS-ATF1 product is required for c-Met expression, and that malignant features such as survival, proliferation, chemotaxis and invasion are dependent on c-Met signaling in cellular models (31). Hence, we may assume that c-Met signaling has probably a connection to the viability of the malignancy.

Current systemic therapies for clear cell sarcoma are of limited benefit (32–36). Tyrosine kinase inhibitors were given in the past to treat patients with CCS in different clinical trials including crizotinib (32, 33), sorafenib (34) tivantinib (35) and sunitinib (33, 36). Generally, responses were poor, partial and very short-lasting with only limited disease stabilization. Early analyses of novel clinical trials investigating sunitinib or chemotherapy in combination with nivolumab are encouraging (37) and clear cell sarcoma will be considered as subgroup in Immunosarc II with further analyses to follow- although stability of disease lasted less 6 months in most cases.

Cabozantinib is an oral small-molecule-inhibitor of multiple tyrosine kinases. Several studies published showed distinct inhibition of MET and VEGFR2, and suppression of metastases, angiogenesis and tumor growth (38–40). The FDA has already approved cabozantinib for renal cell carcinoma (41) and for progressive metastatic medullary thyroid cancer (42). The Children Oncology Group published a phase I study of cabozantinib for resistant solid tumor malignancies (e.g., medullary thyroid carcinoma, wilms tumor, synovial sarcoma), with good tolerability (43). Recently, data emerging in clinical trials evaluating cabozantinib as soft tissue sarcoma treatment, show promising results, with high tolerability of treatment and effective stabilization of the disease (44, 45) and when cabozantinib is combined with immune checkpoint inhibitors (46).

The initial success we had with cabozantinib may have been predictable. The durability of the response is the surprising aspect and the therapeutic achievement of this case. Cabozantinib might prove to be a “game changer” in the treatment approach and the natural history of CCS. The question arises is there contribution of the immunotherapy to the lasting effect of cabozantinib? Early reports imply there is (46). In kidney cancer this combination is now the standard of care. Our experience demonstrates the role of Next Generation Sequencing and molecular diagnosis in enabling targeted therapy in rare forms of solid cancers. Furthermore, it shows that rational prescription of a tyrosine kinase inhibitor based on molecular tumor vulnerability may be significantly augmented by immunotherapy and should be sought in any cancer which lacks good therapeutic options.

## Data availability statement

The raw data supporting the conclusions of this article will be made available by the authors, without undue reservation.

## Ethics statement

Written informed consent was obtained from the individual(s), and minor(s)’ legal guardian/next of kin, for the publication of any potentially identifiable images or data included in this article.

## Author contributions

RSM and GG initiated the case report and substantially contributed to its conception and design, acquisition, analysis and interpretation of the data, and wrote the manuscript. NP, JG, and ML contributed to the acquisition of data and analyses. All authors critically reviewed the manuscript and approved the submitted version.
